# Structural analysis of *Plasmodium falciparum* ookinete surface antigen Pfs28 relevant for malaria vaccine design

**DOI:** 10.1038/s41598-022-24054-3

**Published:** 2022-11-15

**Authors:** Niharika Shukla, Wai Kwan Tang, Niraj H. Tolia

**Affiliations:** Host-Pathogen Interactions and Structural Vaccinology Section, Laboratory of Malaria Immunology and Vaccinology, National Institute of Allergy and Infectious Diseases, National Institutes of Health, Bethesda, USA

**Keywords:** X-ray crystallography, Vaccines, Parasitology

## Abstract

Pfs28 is a *Plasmodium falciparum* malaria transmission-blocking vaccine candidate that is anchored to the parasite surface through a C-terminal glycosylphosphatidylinositol (GPI) moiety, and plays a role in parasite survival in the mosquito midgut. Pfs28 contains epidermal growth factor (EGF)-like domains and is part of a family of sexual stage malaria proteins that includes the related vaccine antigen Pfs25. The lack of structural definition of Pfs28 and the immune response to this candidate has limited further malaria vaccine development for this antigen. Here, we present the crystal structure of Pfs28, examine its conservation with *P. vivax* Pvs28, and evaluate the cross-reactivity of Pfs28 to antibodies that recognize Pfs25. Pfs28 is comprised of four EGF-like domains stabilized by ten disulfide bridges with an overall architecture that highly resembles Pfs25. Despite the high sequence and structural similarity between these antigens, no cross reactivity of Pfs28 to anti-Pfs25 monoclonal antibodies could be demonstrated.

## Introduction

Progress in malaria elimination has slowed, with an estimated 241 million cases and 627,000 deaths caused by the disease in 2020^1^. Advances in malaria treatment and prevention will be necessary to address this profound health burden. RTS,S/AS01, the only World Health Organization-recommended malaria vaccine, targets the pre-erythrocytic stage of the *Plasmodium falciparum* parasite to prevent infection of the human host^1–3^. Transmission-blocking vaccines (TBV) may complement RTS,S by targeting sexual stage parasite development within the mosquito vector, thereby preventing malaria transmission from mosquitoes to humans within a community^4–6^.

Pfs25 is a leading TBV candidate expressed during the sexual stages of the parasite lifecycle and plays a role in ookinete survival in the mosquito midgut as well as traversal through the midgut epithelium^7–10^. Pfs25 has progressed through extensive clinical investigation to assess its safety and efficacy. Several approaches have been explored to increase the immunogenicity of Pfs25, including virus-like particle (VLP) display^11^, nanoparticle display^12^, conjugation to *Pseudomonas aeruginosa* Exoprotein A (EPA)^13,14^, and formulation in diverse adjuvants. Despite these efforts, Pfs25 immunization elicits only moderate transmission-reducing activity (TRA) with rapidly declining antibody titers in humans^12–11^. The structure of Pfs25 has also been solved, and the antibody response to Pfs25 immunization has been characterized structurally and biophysically in efforts to inform structure-based immunogen design^6,15,16^. In conjunction with these approaches to increase and focus the immune response to Pfs25, revisiting the use of Pfs28, another TBV candidate, in individual and combined vaccine approaches may help to progress TBV development.

Pfs28 is a glycosylphosphatidylinositol-anchored protein expressed on the surface of zygotes and ookinetes and plays a similar role as Pfs25 in ookinete survival and traversal through the midgut^17,18^. This function of Pfs28 may be mediated through its interactions with basal lamina proteins surrounding the mosquito midgut, as the *Plasmodium berghei* homolog of Pfs28, has been shown to interact with *Anopheles gambiae* laminin in yeast two-hybrid assays^7^. When Pfs28 is administered as a protein subunit^17,19–21^ or DNA vaccine^22^ in animal models, it can elicit antibodies that inhibit oocyst development when measured in laboratory assays such as the standard membrane feeding assay (SMFA), even achieving complete transmission-blocking activity^17^. Due to the low degree of sequence polymorphism of Pfs28 across *P. falciparum* strains, this antigen may also elicit strain transcending immunity^23,24^.

Pfs28 and Pfs25 are thought to play partially redundant roles in parasite survival within the mosquito, as double knockout *P. berghei* parasites are more impaired in ookinete maturation, midgut epithelium traversal, and oocyst development compared to single knockout or wildtype parasites^10^. Due to the evidence for partially redundant roles for Pfs28 and Pfs25, the efficacy of combined vaccine approaches with both antigens has been evaluated^10^. Mixing sera from mice immunized with the individual antigens leads to synergistic transmission-reducing activity as measured by SMFA^17^. However, it has been challenging to directly elicit this synergistic activity through combined vaccination with both antigens. Depending on the animal model, adjuvant, and antigen construct used, combined Pfs28/Pfs25 vaccines can generate antibody responses with titers and transmission-reducing activity inferior to^25^, similar to^22,26^, or greater than^19^ that of monoantigenic vaccines.

Though a combined Pfs28/Pfs25 vaccine approach holds promise, a greater understanding of the structural and biophysical characteristics of Pfs28 is necessary for structure-based design of immunogens. Here, we report the first crystal structure of Pfs28 and evaluate its structural similarity to Pfs25 and biophysical properties, including its lack of cross-reactivity to a panel of Pfs25-specific monoclonal antibodies (mAbs) and lack of direct interaction to Pfs25. We also evaluate the structural localization of sequence polymorphisms of Pfs28 and its similarity to Pvs28, its *P. vivax* homolog, to provide the basis for the design of broadly protective, strain-transcending, and potentially cross-species immunogens. This work lays the framework for structure-based design of Pfs28/Pfs25 combined vaccines to elicit a more potent immune response.

## Results

### Crystal structure of Pfs28

**Figure 1:**
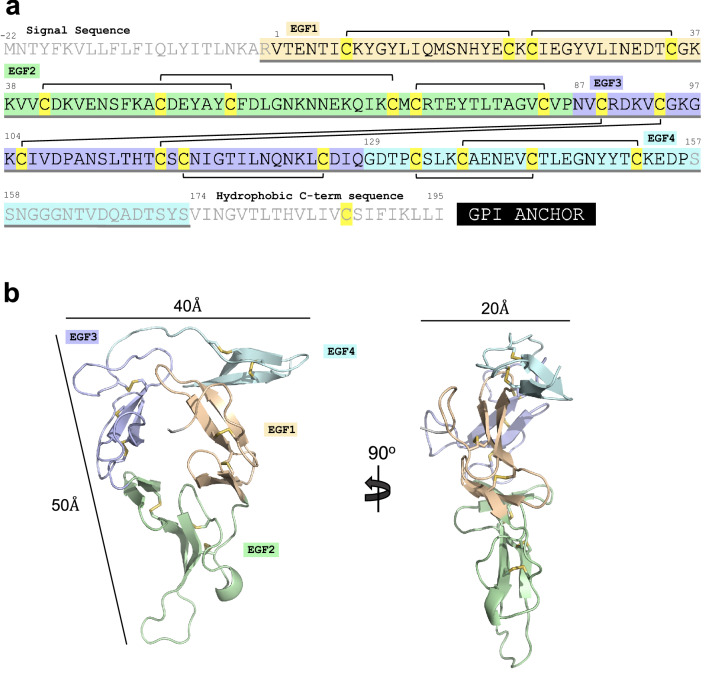
Pfs28 crystal structure. (**a**) Amino acid sequence of Pfs28, depicting N-terminal signal sequence, EGF-like domains 1–4, and the hydrophobic C-terminal sequence that is the site of GPI anchor attachment. Residues are shaded in wheat, green, purple, and blue according to EGF-like domain. Residues included in the construct are underlined in grey, residues that are resolved in the structure are indicated in black, while residues not present in structure are indicated in grey. Cysteines are highlighted in yellow and the disulfide bridging pattern is indicated by black brackets. (**b**) Cartoon representation of Pfs28 with the triangular arrangement of EGF-like domains 1–4 colored wheat, pale green, light purple, and pale cyan and the ten disulfide bridges indicated as yellow sticks.

Pfs28 is a GPI-anchored protein, consisting of an N-terminal signal sequence, four EGF-like domains, and a C-terminal hydrophobic sequence that is the site of GPI attachment. The construct used for crystallography consisted of V1-S173, comprising only the four EGF-like domains (Fig. 1a). The construct was synthesized and cloned into the pHLSec vector with a C-terminal hexahistidine tag for mammalian expression using the Expi293 system as a secreted protein. The recombinant protein was purified using nickel affinity and size exclusion chromatography, then used for structural characterization.

The crystal structure of Pfs28 was solved to a resolution of 2.3 Å (Supplementary Table S1). The Pfs28 structure is comprised of four EGF-like domains in a triangular arrangement, with sides of 40 Å and 50 Å and a thickness of ~ 20 Å, with the overall structure stabilized by 10 disulfide bridges (Fig. 1). Due to flexibility and a lack of clear density at the C-terminus of the protein, the C-terminal portion of EGF4 (residues S157-S173) could not be modelled. EGF domains 1–3 adopt the characteristic structure, consisting of a major two-stranded β-sheet followed by a loop and a shorter two-stranded β-sheet, while only a single two-stranded β-sheet in EGF4 was observed. EGF domains 1 and 4 each have two disulfide bridges, while EGF domains 2 and 3 contain three disulfide bridges (Fig. 1a).

### Sequence similarity between Pfs28 and Pvs28

**Figure 2:**
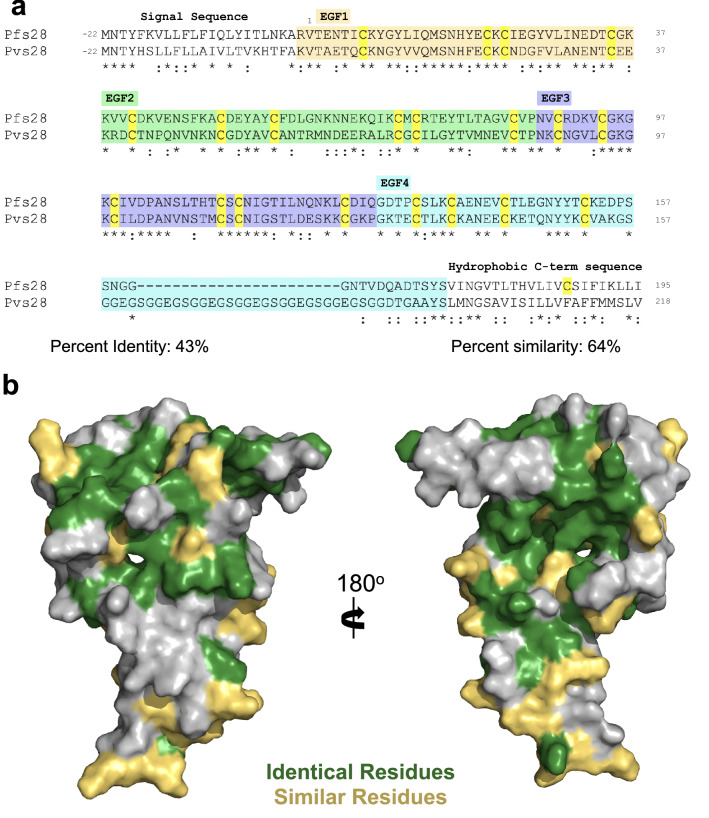
Sequence similarity between Pfs28 and Pvs28. (**a**) Sequence alignment of Pfs28 and Pvs28 generated using Clustal Omega^30,31^ and shaded in wheat, green, purple, and blue according to EGF-like domain. Identical residues indicated with ‘*’ and chemically similar residues indicated with “:” Cysteines are highlighted in yellow. (**b**) Surface representation of Pfs28 (grey) with identical residues colored green and chemically similar residues colored yellow.

*P. vivax* is a significant contributor to malaria morbidity, responsible for 68.3% of cases in the Americas and 36.3% of cases in South-East Asia^1^. Evidence for immune cross-reactivity across *P. falciparum* and *P. vivax* antigens has been reported^27–29^, suggesting potential for a cross-species malaria vaccine. To evaluate whether Pfs28 may elicit cross-species antibodies, sequences and regions of similarity between Pfs28 and its *P. vivax* orthologue, Pvs28, were compared. A sequence identity of 43% and chemical similarity of 64% was determined between both antigens, and the disulfide bridging pattern is highly conserved across all four domains (Fig. 2a). Modelling the identical and chemically similar residues of both antigens on the crystal structure of Pfs28 indicates the majority of identical residues reside within the central region of the protein, at the triangular interface between the EGF domains, in addition to a region of chemically similar residues within EGF2 (Fig. 2b). The major difference between Pfs28 and Pvs28 is that Pvs28 contains a large insertion in the C-terminal region of EGF4. This large insertion is repetitive, consisting of glycine, serine, and glutamate (Fig. 2a).

### Polymorphism analysis of Pfs28

**Figure 3:**
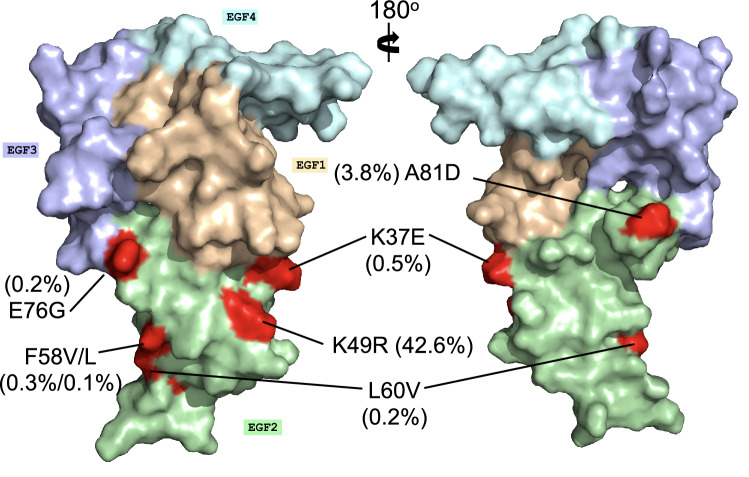
Polymorphism analysis of Pfs28. Surface representation of Pfs28 with EGF 1–4 colored wheat, pale green, light purple, and pale cyan. Polymorphic residues are colored in red and labelled with polymorphic frequencies according to database of 3,488 samples from MalariaGen^32^. Only polymorphisms with frequencies greater than 0.1% are included.

Analysis of 3,488 sequences from MalariaGen^32^ revealed that Pfs28 is conserved across diverse strains of *P. falciparum*. Despite having 50 reported polymorphisms, only 8 of these occur with a frequency higher than 0.1% within the EGF domains of Pfs28 (Fig. 3). These polymorphisms are only found within EGF1, 2, and 4. The polymorphism in EGF4 (A168P), is located within the unstructured C-terminus region of the protein that is not observed. Three polymorphisms occur with frequency greater than 1%: K49R (42.6%), A168P (5.7%), and A81D (3.8%).

### Structural similarity between Pfs28 and Pfs25

**Figure 4:**
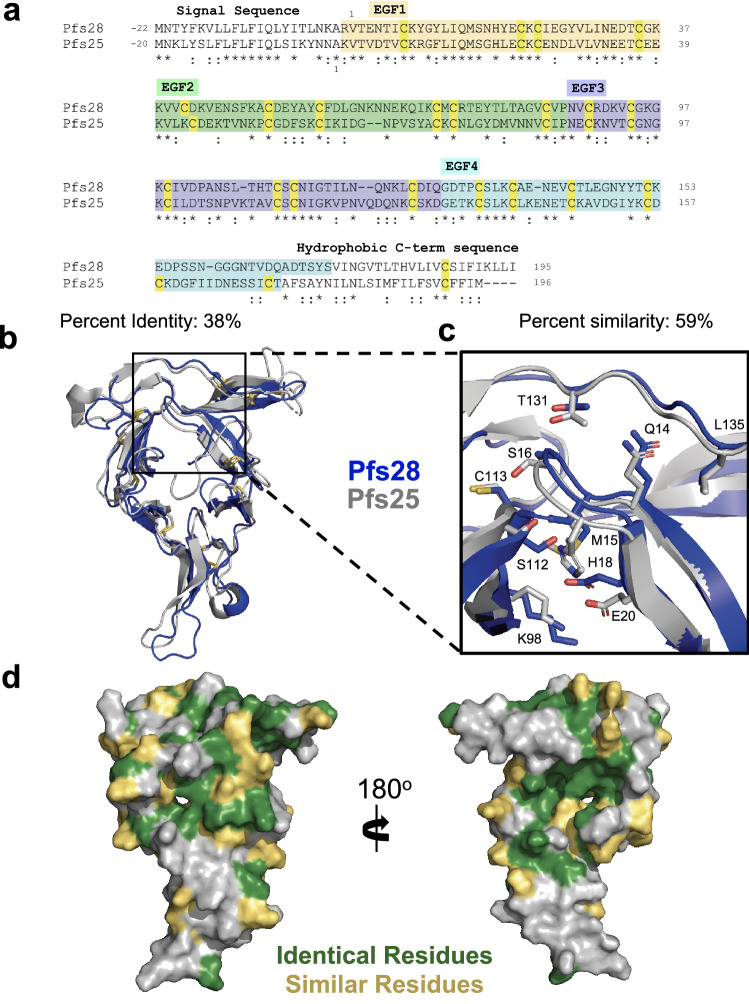
Structural similarity between Pfs25 and Pfs28. (**a**) Sequence alignment of Pfs28 and Pfs25 generated using Clustal Omega^30,31^ and shaded in wheat, green, purple, and blue according to EGF-like domain. Identical residues indicated with ‘*’ and chemically similar residues indicated with “:” Cysteines are highlighted in yellow. (**b**) Superposition of Pfs28 structure (blue) and Pfs25 (grey) (PDB ID: 6PHB)^16^ with disulfide bridges indicated in yellow. (**c**) Detailed views of conserved residues that stabilize triangular arrangement of EGF domains in Pfs25 (PDB ID: 6PHB)^16^ and Pfs28. (**d**) Surface representation of Pfs28 (grey) with identical residues colored green and chemically similar residues colored yellow.

The overall structure of Pfs28 adopts a similar architecture to those of Pfs25 and Pvs25^15,16,33^. The Pfs25 and Pfs28 sequences demonstrate an overall sequence identity of 38%, with 59% chemical similarity (Fig. 4a). The characteristic disulfide bridging pattern of EGF domains is conserved across EGF1-3, though EGF4 of Pfs28 lacks the third disulfide bridge found in Pfs25 (Fig. 4a). Superposition of Pfs28 and Pfs25 (PDB ID: 6PHB)^16^ demonstrates a high degree of structural similarity between both proteins (Fig. 4b), with a root-mean-square deviation (RMSD) of 1.56Å across the C-⍺ atoms of 135 residues. The residues shown to stabilize the triangular arrangement of the EGF domains in Pfs25 are conserved in Pfs28: Q14, M15, S16, H18, E20, K98, S112, C113, T131 and L135 (Fig. 4c). The identical and chemically similar residues between Pfs25 and Pfs28 form large contiguous patches along the central triangular interface of the protein and EGF4 (Fig. 4d).

### Pfs28 does not interact with a panel of anti-Pfs25 mAbs

**Figure 5:**
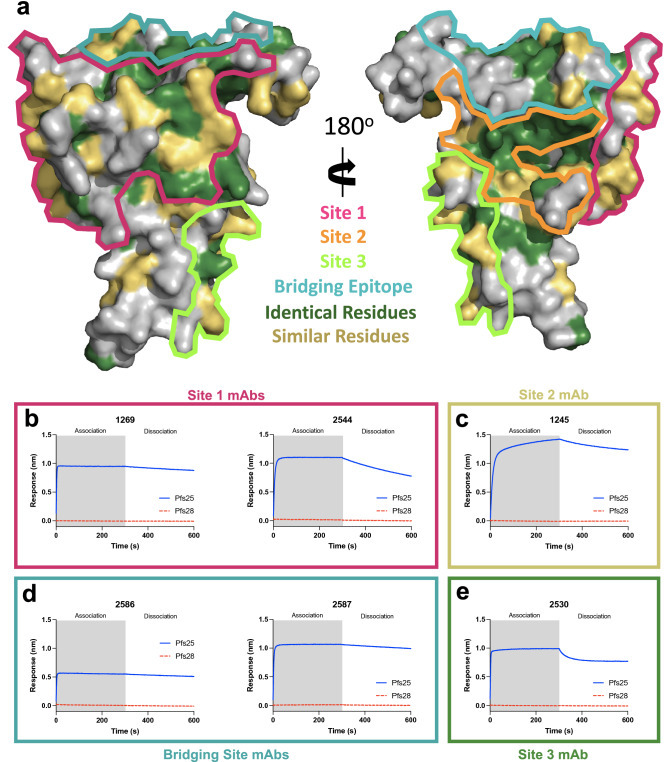
Pfs28 does not cross-react with anti-Pfs25 mAbs. (**a**) Structure of Pfs25 (PDB ID: 6PHB)^16^ with outlined immunogenic sites^15,16^ and identical and chemically similar residues to Pfs28 highlighted in green and yellow. (**b**–**e**) Binding curves of biolayer interferometry experiments using Pfs25 and Pfs28 and transmission reducing Pfs25 mAbs grouped by immunogenic site. Curves represent association and dissociation of each scFv (1 µM) to sensors loaded with Pfs25 (blue) or Pfs28 (red).

The conserved architecture and areas of identical and chemically similar residues between Pfs28 and Pfs25 raise the possibility of conserved epitopes between these two antigens (Fig. 5a). Therefore, Pfs25 monoclonal antibodies (mAbs) were tested for cross reactivity against Pfs28 using biolayer interferometry (BLI). The conserved residues lie within surfaces that are homologous to those previously shown to be the target of transmission-reducing Pfs25 mAbs^15,16^. To probe for cross-reactivity against Pfs28, a panel of human and Kymice-derived monoclonal antibodies with epitopes within Site 1, Site 2, Site 3, and the bridging epitope of Pfs25, as well as a range of transmission reducing activity, were examined (Supplementary Fig. S2a,b)^15,16^. These mAbs were expressed as single chain variable fragments (scFvs) and their association and dissociation to immobilized Pfs28 and Pfs25 were measured at a high concentration of 1 µM to account for the presence of only low affinity interactions with Pfs28. At a concentration of 1 µM of scFv, any background reactivity observed to the sensor alone was subtracted from the raw signal to obtain a measure of the binding to the antigen alone. Though all mAbs showed robust binding to Pfs25, notably, no mAbs demonstrated detectable binding to Pfs28 (Fig. 5b–e).

Examination of the residues within the epitopes of individual Pfs25 mAbs reveals an average of 10 amino acids within each individual epitope that are not identical or chemically similar to those found in Pfs28, with a range from 14 such changes (mAb 1245) to as few as 6 changes (mAb 2544) (Supplementary Fig. S3). For example, within the epitope of mAb 2544, V5 (E3 in Pfs28), G19 (N17), and L21 (Y19) in EGF1 are changed from hydrophobic residues in Pfs25 to charged, polar, or aromatic residues in Pfs28, while T103 (P103) in EGF3 and K135 (P132) in EGF4 are changed from charged or polar residues in Pfs25 to hydrophobic residues in Pfs28. L141 (A148) in EGF4 is changed from a hydrophobic branched chain residue in Pfs25 to a small hydrophobic residue in Pfs28. These nonconservative changes result in the disruption of several hydrophobic interactions as well as a hydrogen bond made between K135 and the light chain of 2544.

### Pfs28 does not interact with Pfs25 by BLI

**Figure 6:**
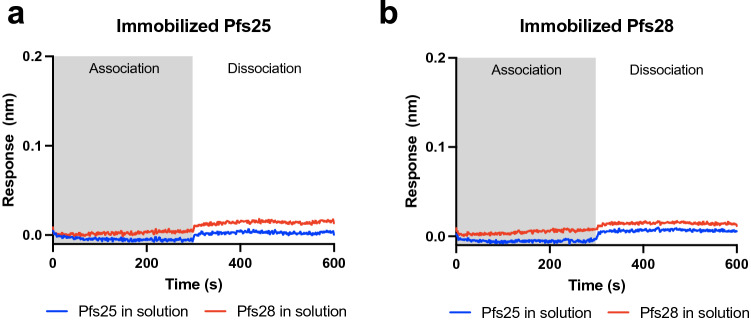
Pfs28 does not interact with Pfs25 by BLI. Binding curves of biolayer interferometry experiments using Pfs25 and Pfs28. Curves represent the association and dissociation of Pfs25 (blue) and Pfs28 (red) (600 nM) to sensors loaded with (**a**) Pfs25 or (**b**) Pfs28.

Pfs28 and Pfs25 have been proposed to directly interact on the surface of parasite in a tile-like fashion^33^. Here, interaction between Pfs25 and Pfs28 was evaluated using BLI. When either Pfs25 or Pfs28 were immobilized on the sensor in separate assays, no interaction with either antigen at a concentration of 600 nM in the buffer was detectable (Fig. 6a,b). This result suggests Pfs28 and Pfs25 cannot form an interaction when immobilized on a pin. A limitation of this result is the GPI-anchoring of both proteins on the parasite surface may facilitate an interaction not replicable using BLI.

## Discussion

Effective malaria transmission-blocking vaccine approaches will be key to reigniting progress towards malaria elimination and eradication. Though Pfs25 has been one of the leading TBV candidates, recent clinical trials have achieved only modest efficacy^12–14^. Additional antigens and approaches are needed, and combined vaccine approaches which include Pfs28 may induce synergistic transmission-reducing antibodies that can help overcome this hurdle^17^, though it has been challenging to directly elicit this synergistic antibody response through vaccination with combined Pfs28/Pfs25 antigens^25^. Pfs25 and its epitopes for transmission-reducing mAbs have been extensively characterized structurally, and a similar degree of understanding of Pfs28 will be necessary for structure-based design of future TBVs^15,16^. In this study, we describe the crystal structure of Pfs28 and show its high resemblance to Pfs25. Despite this similarity between Pfs28 and Pfs25, we did not observe any interaction of Pfs28 with Pfs25 transmission-reducing mAbs.

The structure of Pfs28 adopts a triangular arrangement of its four EGF-like domains. The majority of the Pfs28 surface is highly conserved across *P. falciparum* strains, with only three polymorphisms occurring with a frequency greater than 1%. These polymorphisms are limited to EGF2 and 4 and suggest that immunization with Pfs28 may elicit broadly protective immunity. The triangular arrangement in Pfs28 is similar to what is seen in Pfs25, with conservation of key residues shown to promote the triangular assembly^15,16,33^. The major difference between the structures of the two antigens lies in the lack of the third disulfide bridge in EGF4 of Pfs28. This is reflected in the greater degree of disorder present in EGF4 of Pfs28 compared to Pfs25, such that its C-terminal region could not be resolved in the crystal structure. This contrasts previous crystal structures of Pfs25 which have shown a clearly resolved conformation of the C-terminus portion of EGF4^15,16^. The physiological importance of this greater flexibility at the C-terminus, and how it is involved in the function of Pfs28 (or its antibody-mediated neutralization), must be further explored.

A tile-like pattern of crystal packing has been observed previously in the structure of Pvs25, and suggested a role for Pfs25 and Pfs28 and their homologs in forming protective, tiled sheets on the parasite surface^33^. However, the crystal packing of Pfs28 did not adopt a similar tile-like pattern and we also saw no evidence of interaction between Pfs25 and Pfs28 as measured by BLI. Our results agree with the findings of Scally et al.^15^ who showed the epitopes of Site 1 Pfs25 mAbs are inaccessible in the tiled model, and that the model was thus inconsistent with high transmission blocking activity observed for these mAbs. The arrangement of Pfs25 and Pfs28 on the parasite surface remains uncharacterized, and future work to define this arrangement may provide insight into key surfaces that can be targeted to disrupt physiologically important interactions.

Beyond the conserved global architecture of Pfs25 and Pfs28, we also observed a high degree of sequence identity (38%) and chemically similar (59%) residues across the two antigens. These areas of similarity formed large, contiguous patches on the surface of Pfs28 that aligned with homologous surfaces on Pfs25 that are targeted by transmission-reducing mAbs^15,16^. Despite the similarity of both antigen surfaces, there was no evidence of cross-reactivity of Pfs25 mAbs across four immunogenic sites as measured by BLI. These results are consistent with the findings of Zaric et al.^34^, which demonstrated that polyclonal sera raised against Pfs25 did not show cross-reactivity against Pfs28. Though the structure of Pvs28 is still uncharacterized, comparison of its sequence to Pfs28 showed 43% sequence identity. Cross-species reactivity of antibody between Pfs28 and Pvs28 remains unknown.

Combined vaccine approaches have shown promise with HIV^35^, influenza^36^, *Neisseria meningitidis*^37^, and SARS-CoV-2^38,39^ antigens to elicit antibodies that target functional surfaces from multiple antigens simultaneously, or to focus the immune response away from non-neutralizing, immunodominant surfaces. These findings lay the framework for the structure-based design of combined Pfs28/Pfs25 immunogens. Future studies to structurally, biophysically, and functionally characterize the antibody response to Pfs28 vaccination will further reveal the mechanisms of antibody-mediated neutralization of Pfs28, and inform which surfaces should be prioritized in combined vaccine approaches.

## Materials and methods

### Expression and purification of Pfs28, Pfs25 and scFvs

Protein sequences for Pfs25 (PF3D7_1031000) and Pfs28 (PF3D7_1030900) were obtained from PlasmoDB. The wildtype Pfs28 sequence was used, but the Pfs25 construct used for biophysical studies contained three N-linked glycosylation sites at residues 91, 144, and 166 that were mutated from asparagine to glutamine. Single chain variable fragments (scFvs) of mAbs were designed by fusing the V_H_ region of each mAb to its paired V_L_ region by a (GGGGS)_4_ linker.

All constructs were cloned into the pHLsec plasmid with a C-terminus hexa-histidine tag and transiently expressed in Expi293 cells following manufacturer protocol (Thermo Fisher Scientific, Waltham, MA) as secreted protein then harvested four to five days after transfection. After centrifugation, the supernatant was loaded on Ni Sepharose Excel resin (Cytiva, Marlborough, MA) and washed with 10 column volumes of wash buffer (25 mM Tris pH 7.4, 300 mM NaCl, 30 mM imidazole). Recombinant protein was eluted with five column volumes of elution buffer (25 mM Tris pH 7.4, 300 mM NaCl, 150 mM imidazole) and concentrated using an Amicon Ultra Centrifugal filter with 10 kDa molecular weight cutoff (Millipore Sigma, Burlington, MA). Concentrated eluate was purified by size exclusion chromatography using a Superdex 75 Increase 10/300 GL column (Cytiva, Marlborough, MA) equilibrated in 20 mM Tris pH 8.0, 100 mM NaCl.

### Crystallization and structure determination

Purified Pfs28 was concentrated to 10 mg/ml before use in crystallization experiments.

The protein solution was mixed in a 1:1 ratio with mother liquor and used to set up crystallization trays at 18 °C. Crystals of Pfs28 were grown in 0.5 M ammonium sulfate and 30% PEG 4000 using hanging-drop vapor diffusion and cryoprotected in 30% PEG 400 before flash freezing in liquid nitrogen.

X-ray diffraction data were collected at the SER-CAT 22ID beamline at the Advanced Photon Source, Argonne National Laboratory. Data were processed and scaled using XDS^40^. Molecular replacement was performed in PHASER^41^ using an AlphaFold2 Pfs28 model (AF-Q8IJ96-F1) as a search model^42,43^. Initial models were built using PHENIX AutoBuild and iterations of model building and refinement were carried out using COOT^44^ and PHENIX^41^.

### Protein–protein interaction studies using biolayer interferometry (BLI)

All BLI experiments were performed using the OctetRED 96 (Sartorius, Fremont, CA) at 25 °C. Pfs28 and Pfs25 were biotinylated in vivo at a C-terminus Avi-Tag encoded in the pHL-AviTag3 plasmid (a gift from E.Y. Jones)^45^ in Expi293 cells by transfection with biotin ligase BirA expressing plasmid (a gift from Daved Fremont, Washington University, St. Louis, MO) in a 9:1 ratio. The media contained biotin at a concentration of 100 μM. After purification, all scFvs were buffer exchanged into HBS-EP + buffer (Cytiva, Marlborough, MA) using Zeba Microspin Desalting columns with a 7 kDa molecular weight cutoff (Thermo Fisher Scientific, Waltham, MA). Pfs28 and Pfs25 were then diluted to 10 nM and all scFvs were diluted to 1 µM in HBS-EP + buffer.

Biotinylated Pfs25 and Pfs28 were loaded on streptavidin biosensors (Sartorius, Fremont, CA) for 300 s and a baseline measurement was obtained for 60 s. The loaded sensors were dipped into wells containing each scFv (1 µM) to measure association and then dipped into a well containing buffer to measure dissociation. Both steps were 300 s long.

Data were analyzed using Octet Data Analysis HT 12.0 Software (Sartorius, Fremont, CA). Double reference subtraction was carried out to subtract binding signal of unliganded sensor in 1 µM of scFv and of antigen loaded sensors in buffer alone from individual binding curves.

### Ethics statement

No animals or human clinical samples were used in this study.

## Supplementary Information

Supplementary Information.

## Data Availability

Atomic coordinates and structure factors have been deposited in the Protein Data Bank with the accession code 8E1Z. All other data generated or analyzed during this study are included in this published article (and its supplementary information files).
