# Supplementary material for: Structural analysis of Plasmodium falciparum ookinete surface antigen Pfs28 relevant for malaria vaccine design
Source: Sci Rep. 2022 Nov 15;12:19556. doi: 10.1038/s41598-022-24054-3 (PMC9664031; doi:10.1038/s41598-022-24054-3)
Supplement: Supplementary file 1 — Supplementary Information. [file 41598_2022_24054_MOESM1_ESM.pdf]

Supplementary Information for

Structural analysis of *Plasmodium falciparum* ookinete surface antigen Pfs28 relevant for malaria vaccine design

Niharika Shukla, Wai Kwan Tang, Niraj H. Tolia

*Corresponding author: niraj.tolia@nih.gov

Contents:

Supplementary Figures S1-S3
Supplementary Table S1

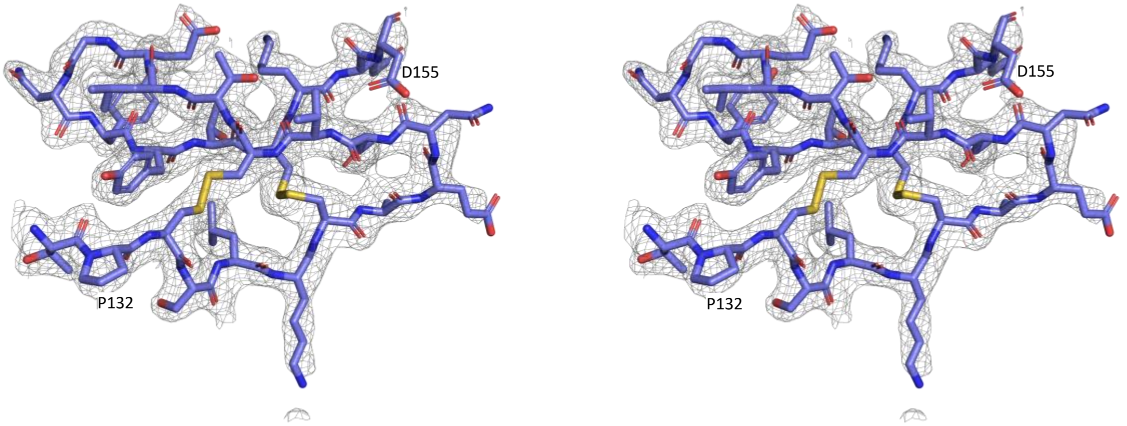

Supplementary Figure S1: Stereo view of representative electron density of Pfs28.
Pfs28 shown in blue. 2Fo-Fc electron density map contoured in grey at 1σ.

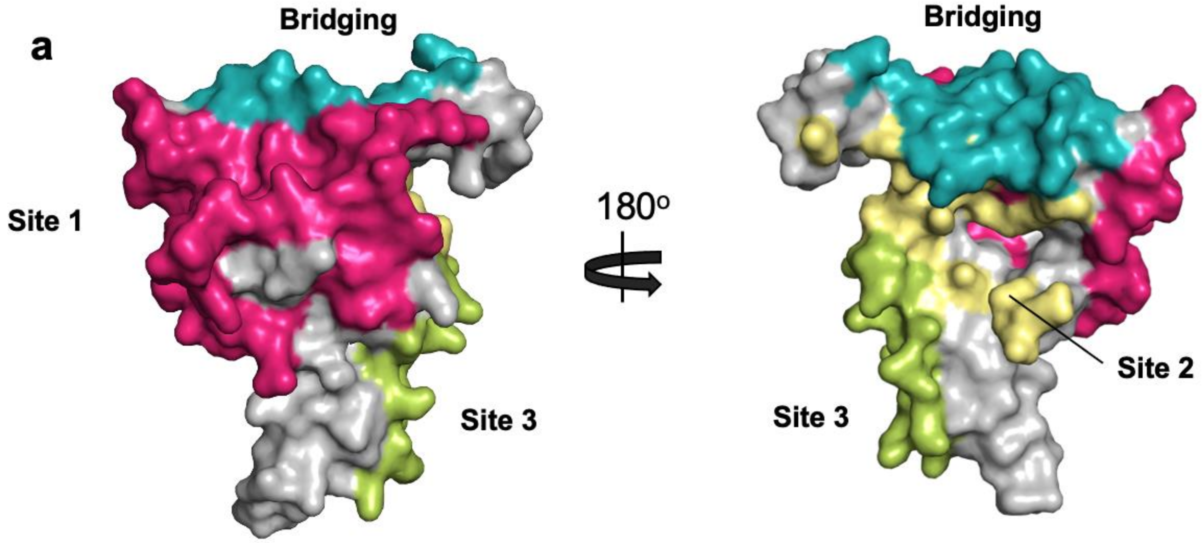

b

Name	Epitope	K_D (nM)	%TRA at 10 μg/ml	%TRA at 100 μg/ml	%TRA at 375 μg/ml	IC80 (μg/ml)
2544	Site 1	4.6 ± 1.2	-	100	-	16
1269	Site 1	3.7 ± 0.3	-24.4	-	100	63
2586	Bridging	2.5 ± 0.2	-	92.2	-	96
2587	Bridging	4.6 ± 2.5	-	84.5	-	-
1245	Site 2	31.0 ± 5.6	-39.5	-	93.2	263
2530	Site 3	19.2 ± 1.9	-	88.3	-	65

Supplementary Figure S2: Epitopes and transmission-reducing activity of Pfs25 mAbs used in cross-reactivity experiments. a. Surface representation of Pfs25 (PDB ID: 6PHB) colored by immunogenic site [11,19]. **b.** Transmission reducing activity of Pfs25 mAbs and binding affinity of respective antibody fragment antigen-binding region (Fab) [11,19].

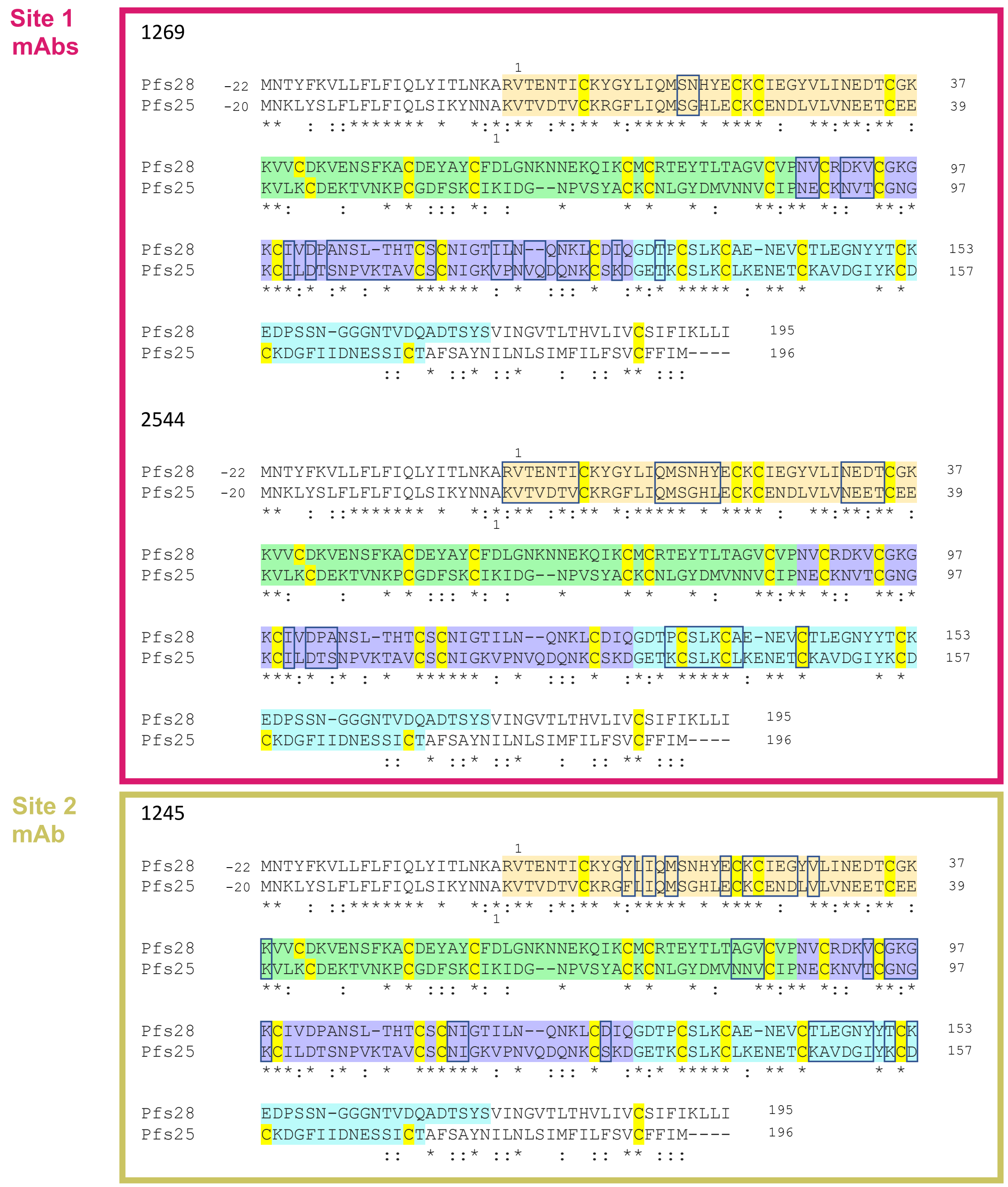
Site 1 mAbs
1269
1
Pfs28 -22 MNTYFKVLLFLFIQLYITLNKARVTENTICKYGYLIQMSNHYECKCIEGYVLINEDTCGK 37
Pfs25 -20 MNKLYSLFLFLFIQLSIKYNNAKVTVDTVCKRGFLIQMSGHLECKCENDLVLVNEETCEE 39
** : ::******* * *:*:** :*:** *:***** * **** : **:**:** :
1
Pfs28 KVVCDKVENSFKACDEYAYCFDLGNKNNEKQIKCMCRTEYTLTAGVCVPNVCRDKVCGKG 97
Pfs25 KVLKCDEKTVNKPCGDFSKCIKIDG--NPVSYACKCNLGYDMVNNVCIPNECKNVTCGNG 97
: : * * ::: *: : * * * * : **: *:: **:*
Pfs28 KCIVDPANSL-THTCSCNIGTILN--QNKLCDIQGDTPCSLKCAE-NEVCTLEGNYYTCK 153
Pfs25 KCILDTSNPVKTAVCSCNIGKVPNVQDQNKCSKDGETKCSLKCLKENETCKAVDGIYKCD 157
:* :* : * *** : * ::: * :*:* ***** : ** * * *
Pfs28 EDPSSN-GGGNTVDQADTSYSVINGVTLTHVLIVCSIFIKLLI 195
Pfs25 CKDGFIIDNESSICTAFSAYNILNLSIMFILFSVCFFIM---- 196
:: * ::* ::* : :: ** :::
2544
1
Pfs28 -22 MNTYFKVLLFLFIQLYITLNKARVTENTICKYGYLIQMSNHYECKCIEGYVLINEDTCGK 37
Pfs25 -20 MNKLYSLFLFLFIQLSIKYNNAKVTVDTVCKRGFLIQMSGHLECKCENDLVLVNEETCEE 39
** : ::******* * *:*:** :*:** *:***** * **** : **:**:** :
1
Pfs28 KVVCDKVENSFKACDEYAYCFDLGNKNNEKQIKCMCRTEYTLTAGVCVPNVCRDKVCGKG 97
Pfs25 KVLKCDEKTVNKPCGDFSKCIKIDG--NPVSYACKCNLGYDMVNNVCIPNECKNVTCGNG 97
: : * * ::: *: : * * * * : **: *:: **:*
Pfs28 KCIVDPANSL-THTCSCNIGTILN--QNKLCDIQGDTPCSLKCAE-NEVCTLEGNYYTCK 153
Pfs25 KCILDTSNPVKTAVCSCNIGKVPNVQDQNKCSKDGETKCSLKCLKENETCKAVDGIYKCD 157
:* :* : * *** : * ::: * :*:* ***** : ** * * *
Pfs28 EDPSSN-GGGNTVDQADTSYSVINGVTLTHVLIVCSIFIKLLI 195
Pfs25 CKDGFIIDNESSICTAFSAYNILNLSIMFILFSVCFFIM---- 196
:: * ::* ::* : :: ** :::
Site 2 mAb
1245
1
Pfs28 -22 MNTYFKVLLFLFIQLYITLNKARVTENTICKYGYLIQMSNHYECKCIEGYVLINEDTCGK 37
Pfs25 -20 MNKLYSLFLFLFIQLSIKYNNAKVTVDTVCKRGFLIQMSGHLECKCENDLVLVNEETCEE 39
** : ::******* * *:*:** :*:** *:***** * **** : **:**:** :
1
Pfs28 KVVCDKVENSFKACDEYAYCFDLGNKNNEKQIKCMCRTEYTLTAGVCVPNVCRDKVCGKG 97
Pfs25 KVLKCDEKTVNKPCGDFSKCIKIDG--NPVSYACKCNLGYDMVNNVCIPNECKNVTCGNG 97
: : * * ::: *: : * * * * : **: *:: **:*
Pfs28 KCIVDPANSL-THTCSCNIGTILN--QNKLCDIQGDTPCSLKCAE-NEVCTLEGNYYTCK 153
Pfs25 KCILDTSNPVKTAVCSCNIGKVPNVQDQNKCSKDGETKCSLKCLKENETCKAVDGIYKCD 157
:* :* : * *** : * ::: * :*:* ***** : ** * * *
Pfs28 EDPSSN-GGGNTVDQADTSYSVINGVTLTHVLIVCSIFIKLLI 195
Pfs25 CKDGFIIDNESSICTAFSAYNILNLSIMFILFSVCFFIM---- 196
:: * ::* ::* : :: ** :::

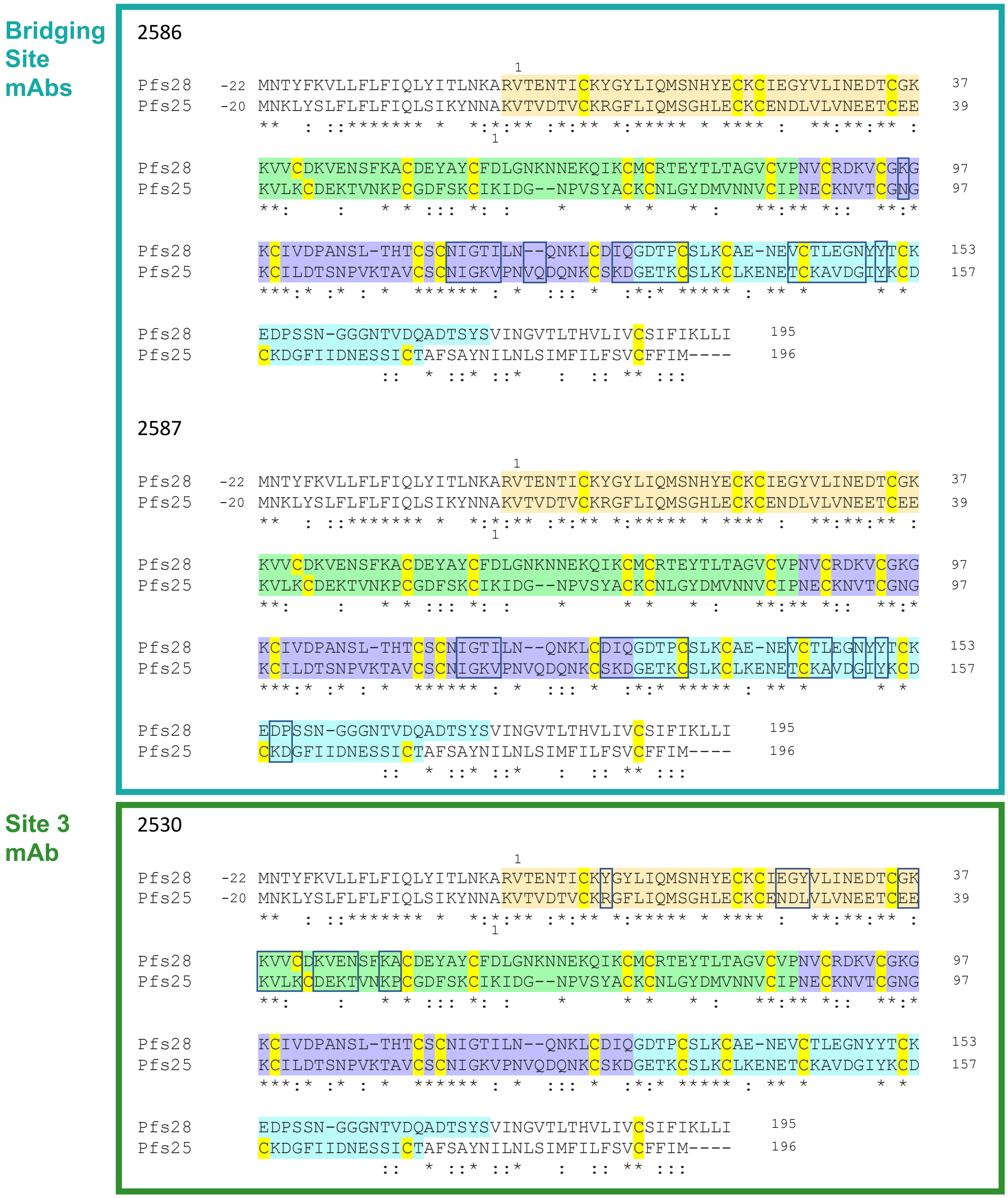

Supplementary Figure S3: Conserved residues within Pfs25 mAb epitopes. Sequence alignment of Pfs28 and Pfs25 generated using Clustal Omega [44,45] and shaded in wheat, green, purple, and blue according to EGF-like domain depicting N-terminal signal sequence, EGF domains 1-4, and hydrophobic C-terminal sequence. Residues are highlighted in wheat, green, purple, and blue according to EGF-like domain, while N-terminal signal sequence and C-terminal sequence are not highlighted. Residues that are identical are indicated by ‘*’ while residues that are chemically similar are indicated by ‘:’. Residues comprising the epitopes of each Pfs25 mAb are indicated by a dark blue box.

**Wavelength**	1.00
**Resolution range**	34.59 - 2.3 (2.382 - 2.3)
**Space group**	P 1 21 1
**Unit cell**	35.73 74.18 69.82 90 105.17 90
**Total reflections**	52298 (5430)
**Unique reflections**	15434 (1579)
**Multiplicity**	3.4 (3.4)
**Completeness (%)**	97.73 (98.50)
**Mean I/sigma(I)**	7.02 (1.98)
**Wilson B-factor**	40.47
**R-merge**	0.1181 (0.676)
**R-meas**	0.1408 (0.8032)
**R-pim**	0.07575 (0.4288)
**CC1/2**	0.991 (0.772)
**CC***	0.998 (0.933)
**Reflections used in refinement**	15392 (1577)
**Reflections used for R-free**	1528 (162)
**R-work**	0.2336 (0.3031)
**R-free**	0.2761 (0.3478)
**CC(work)**	0.933 (0.776)
**CC(free)**	0.894 (0.684)
**Number of non-hydrogen atoms**	2444
**macromolecules**	2406
**ligands**	6
**solvent**	32
**Protein residues**	314
**RMS(bonds)**	0.003
**RMS(angles)**	0.57
**Ramachandran favored (%)**	95.16
**Ramachandran allowed (%)**	4.84
**Ramachandran outliers (%)**	0.00
**Rotamer outliers (%)**	0.35
**Clashscore**	1.06
**Average B-factor**	51.91
**macromolecules**	52.08
**ligands**	47.16
**solvent**	39.35

Supplementary Table S1: Crystallographic data collection and refinement statistics
